# Potential Pathophysiological Mechanisms Underlying Multiple Organ Dysfunction in Cytokine Release Syndrome

**DOI:** 10.1155/2022/7137900

**Published:** 2022-04-06

**Authors:** Peixian Chen, Yan Tang, Weixin He, Ruixuan Yang, Zhien Lan, Ruirong Chen, Peidong Zhang

**Affiliations:** ^1^Zhujiang Hospital, Southern Medical University/the Second School of Clinical Medicine, Southern Medical University, 253 Industrial Avenue, Guangzhou, 510282 Guangdong, China; ^2^Nanfang Hospital, Southern Medical University/the First School of Clinical Medicine, Southern Medical University, No. 1023, South Shatai Road, Baiyun District, Guangzhou, 510515 Guangdong, China; ^3^Affiliated South China Hospital, Southern Medical University (Guangdong Provincial People's Hospital), No. 106, Zhongshan 2nd Road, Yuexiu District, Guangzhou, 510055 Guangdong, China; ^4^Department of Cardiology, Heart Center, Zhujiang Hospital, Southern Medical University, 253 Industrial Avenue, Guangzhou, 510282 Guangdong, China

## Abstract

In recent decades, many serious respiratory infections have broken out all over the world, including SARS-CoV, MERS, and COVID-19. They are characterized by strong infectivity, rapid disease progression, high mortality, and poor prognosis. Excessive immune system activation results in cytokine hypersecretion, which is an important reason for the aggravation of symptoms, and can spread throughout the body leading to systemic multiple organ dysfunction, namely, cytokine release syndrome (CRS). Although many diseases related to CRS have been identified, the mechanism of CRS is rarely mentioned clearly. This review is intended to clarify the pathogenetic mechanism of CRS in the deterioration of related diseases, describe the important signaling pathways and clinical pathophysiological characteristics of CRS, and provide ideas for further research and development of specific drugs for corresponding targets to treat CRS.

## 1. Introduction

Cytokine release syndrome (CRS), as is known to all, is an uncontrollable systemic inflammatory response syndrome (SIRS) caused by excessive release of cytokines. It causes a series of clinical symptoms due to cytokine storms attacking multiple systems and organs. In critically ill patients, they can evolve into multiple organ failure, even be life-threatening. A variety of grading methods have been proposed for CRS. The severity of CRS can be roughly divided into four levels: mild, moderate, severe, and life-threatening [1–4]. Patients with mild CRS mainly show nonspecific clinical symptoms such as fever, rash, fatigue, anorexia, diarrhea, joint pain, headache, myalgia, and neuropsychiatric symptoms, while those with severe CRS may have symptoms of multiple organ failure, such as cognitive impairment, respiratory distress, and shock [5]. A survey found that nearly half of all patients diagnosed with CRS had severe CRS and a poor prognosis. Therefore, the severe form of CRS warrants urgent research attention.

CRS involves cascade activation of circulating cytokines and hyperactivation of immune cells, which can be triggered by infection, malignant tumors, autoimmunity, monogenic disease, various treatments, and some drugs [5, 6]. In recent decades, an increasing number of diseases have been found to be associated with CRS [7]. Scientists first discovered and described CRS in acute graft versus host disease, which is induced by the transplantation of allogeneic hematopoietic stem cells [8]. Subsequently, CRS was also found in patients receiving treatment of immune checkpoint inhibitors or chimeric antigen receptor (CAR) T cells [9]. During the course of therapy, the severity of CRS and neurotoxicity was found to have a direct dependency between the burden of acute lymphocytic leukemia (ALL) and the extent of CAR T cell expansion [10, 11]. Furthermore, CRS has been observed in patients with primary and secondary hemophagocytic lymphohistiocytosis (HLH), macrophage activation syndrome (MAS), and SARS-COV-, MERS-, or COVID-19-induced acute respiratory distress syndrome (ARDS) [12, 13].

Until recently, COVID-19 has spread throughout many countries all over the world for nearly two years. Nevertheless, the number of confirmed cases and the death toll continue to rise, causing panic among governments and people all over the world. What relentlessly takes the patient's life is the overactive immune response induced by SARS-CoV-2 virus infection. This response involves the release of extensive proinflammatory cytokines, resulting in a violent inflammatory response [14]. CRS occurs when systems and organs throughout the body are attacked by inflammatory cytokines. Patients with severe lung injury can progress to ARDS [15, 16]. The obvious hypoxemia that accompanies it puts the patient at risk. It can be concluded that CRS may be a common pathophysiological syndrome in the occurrence and progression of various diseases. Unfortunately, scientists have yet to find an effective treatment for CRS. We provide an overview of the fundamental mechanisms that lead to multiple organ damage in CRS, in order to identify the key cytokines or common pathways involved in the initiation and development of CRS and what pathophysiological damage these cytokines cause to organs. Based on these, we are dedicated to find corresponding therapeutic drugs to explore mutual therapeutic treatment of diseases involving CRS, which will have a beneficial impact on the prevention and treatment of multiple serious diseases [17].

We searched for published articles in PubMed and Google Scholar, employing the keywords “cytokine storm” and “cytokine release syndrome” and discovered that they are associated with multiple diseases, such as COVID-19, acute graft versus host disease, and MERS. We also summarize cytokine interaction network and identify that four cytokines play a major role in the development of CRS by analyzing those publications combining CRS and relevant disorders. After reading literature intensively, it was found that CRS can cause multiple organ injuries, especially brain, lung, heart, liver, and kidney injuries. Therefore, we conducted a further search using the terms “organ injury” and “cytokine” to summarize the cytokines and pathways involved in CRS-related organ damage. Finally, we took “COVID-19” as pointcut and searched for inhibitors of relevant cytokines or pathways to explore effective treatment of CRS.

## 2. Common Features of Cytokine Storms

The analysis of clinical case shows that the clinical symptoms of CRS in different diseases have shared characteristics. Cytokine storms can assault various systems and organs throughout the body and generally manifest as lung, kidney, or liver dysfunction. When cytokine storms affect the lung, mild symptoms can include cough and shortness of breath. With progression of the disease, not only pneumonia but also pulmonary edema can occur. When the condition worsens, CRS is able to develop into ARDS, along with dyspnea and hypoxemia [5, 6, 18]. When the kidney is involved, it presents as proteinuria, acute kidney injury (AKI), and renal dysfunction [19–21]. When the liver is involved, CRS is characterized by hepatomegaly, elevation of liver enzymes and hypoproteinemia, cholestasis, liver injury, and even liver failure [5].

Other systems may also be affected. Damage to the heart caused by CRS leads to hypotension, arrhythmia, cardiomyopathy, and even heart failure (HF) [22–24]. Nervous system damage manifests as dizziness, muscle pain, confusion, ataxia, epilepsy, loss of taste and smell, and defects in visual acuity [25]. Injury to blood vessels can lead to capillary leakage syndrome, hemodynamic instability, and coagulation activation [26, 27], while gastrointestinal symptoms include loss of appetite, nausea, vomiting, and diarrhea [28–30].

**Figure 1:**
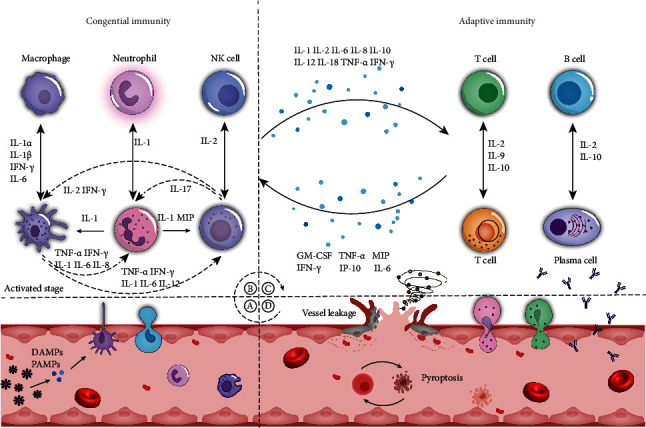
Development of cytokine release syndrome. (a) Antigen presentation: when DAMPs (damage-associated molecular patterns) or PAMPs (pathogen-associated molecular patterns) bind to PRRs (pattern recognition receptors) on the membrane of antigen-presenting cells (APCs) in capillaries, these cells activate immune signal transduction and stimulate immune cells and epithelial cells to release cytokines. (b) Activation of the innate immune system: cytokine secretion results in the recruitment of innate immune cells (e.g., macrophages, neutrophils, and NK cells) in local tissues. (c) Activation of adaptive immune system: innate immune cells further activate adaptive immune cells (T cells and B cells). Immune cells then continue to activate each another, resulting in extensive production of cytokines under the action of positive feedback and causing the formation of a cytokine storm. (d) Cytokine release syndrome: after a cytokine storm is formed, immune cells and the cytokines released by them continue to induce capillary leakage and pyroptosis, which can lead to severe organ structural destruction and functional failure. IP-10: interferon-inducible protein 10; MIP: macrophage inflammatory protein; GM-CSF: granulocyte-macrophage colony-stimulating factor; IL: interleukin; TNF-*α*: tumor necrosis factor-*α*; IFN-*γ*: interferon-*γ*; NK cells: natural killer cells.

At the molecular level, we found that different diseases trigger cytokine storms by different initiation mechanisms and variations in the spectrum of cytokines that become activated [31–34]. Nevertheless, CRS has shared characteristics in regard to injury to different organs: cytokines induce cell injury and endothelial dysfunction, leading to vascular leakage and nonprogrammed cell death eventually (Figure 1). Through repeated comparison, we found that several cytokines, including IL-1 (interleukin-1), IL-6 (interleukin-6), TNF-*α* (tumor necrosis factor-*α*), and IFN-*γ* (interferon-*γ*), always coexist and are critical to CRS caused by infection or immunotherapy [35]. These cytokines are initially produced primarily in innate immune cells such as monocytes, macrophages, neutrophils, and natural killer (NK) cells. Innate immune cells not only activate each other but also release cytokines to activate adaptive immune cells, which further aggravates the inflammatory effect. After activation, adaptive immune cells in turn act on innate immune cells and positively upregulate cytokine levels. When exposure to the external stimulus is continuous, positive feedback always exists while the negative feedback mechanism is insufficiently activated. This cascade amplification and continuous positive feedback bring about the overactivation of the immune system as well as the aggregation of immune cells in local tissues, which stimulates the manufacture of free radicals and proteases, which can directly damage cells, tissues, and organs. These mechanisms ultimately cause a development of CRS.

Next, this review summarizes the mechanisms underlying the downstream effects caused by the common and crucial cytokines in CRS and attempts to explain how they lead to inflammatory damage to vital organs, that is, how CRS develops. The insights thus gained should provide inspiration for developing new treatments against CRS.

## 3. Cytokine Signaling Pathway Activated by IL-6, TNF-*α*, IFN-*γ*, IL-1, and the Inflammasome

**Figure 2:**
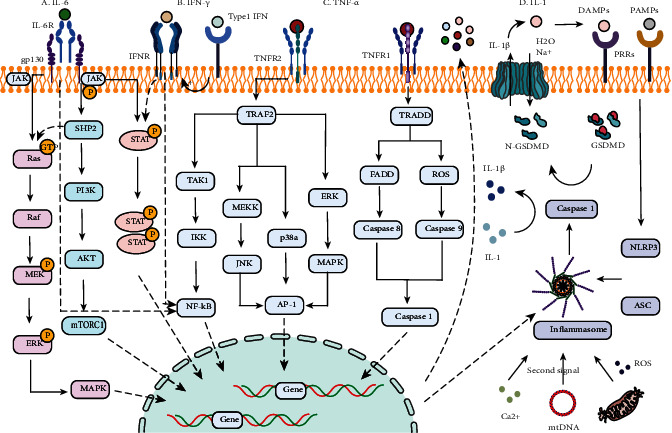
Cytokine signaling pathway activated by IL-6, TNF-*α*, IFN-*γ*, IL-1, and the inflammasome. (a) IL-6 mainly mediates signal transduction through Ras-Raf-ERK-MAPK, NF-*κ*B, JAK/STAT3, and PI3K-Akt-mTOR. (b) IFN-*γ* activates the downstream pathway through JAK/STAT1 and NF-*κ*B to generate a cascade amplification effect. (c) TNF-*α* promotes inflammatory response diffusion through NF-*κ*B activation (activated by the sequential recruitment of TRAF2, RIP, TAK1, and IKK) and induces apoptosis by activation of AP-1 and caspase-3. After recruitment of TRAF2 and RIP, AP-1 can be activated through three pathways, in which the following key molecules are involved: ① MEKK and JNK, ② P38a, and ③ ERK and MAPK. Caspase-3 levels can be elevated by sequential recruitment of TRADD, FADD, and caspase-8 or via TRADD, ROS, and caspase-9. (d) IL-1 decomposes into IL-1*α* and IL-1*β* under inflammasome activation, and then, IL-1*β* is released through Gasdermin D (GSDMD) lysate pore and induces cell swelling and apoptosis. The first and second signals are necessary for activation of the inflammasome. JAK: Janus kinase; STAT: the signal transducer and activator of transcription; Ras: a GTPase; Raf: Raf kinase; ERK: extracellular signal-regulated kinase; MAPK: mitogen-activated protein kinase; MEK: MAPK/ERK kinase; SHP2: Src homology 2-containing protein tyrosine phosphatase 2; PI3K: phosphatidylinositol 3 kinase; AKT: protein kinase B (PKB); mTORC1: mammalian target of rapamycin complex 1; TRAF2: TNFR-associated factor 2; RIP: receptor-interacting protein; TAK1: TGF-*β*-activated kinase 1; TGF-*β*: transforming growth factor-*β*; IKK: inhibitor of *κ*B kinase; MEKK: MAP/ERK kinase kinase; JNK: c-Jun N-terminal kinase; p38a: p38 mitogen-activated protein kinase; AP-1: activator protein-1; TRADD: TNFR-associated death domain; FADD: Fas-associated protein with death domain; NLRP3: NOD-like receptor (NLR) protein 3; ASC: adaptor protein apoptosis-associated speck-like protein containing a caspase-recruitment domain; caspase: cysteinyl aspartate specific proteinase.

After binding to their corresponding receptors, IL-6, TNF-*α*, IFN-*γ*, and IL-1 affect the expression of cytokine-related genes in the nucleus through transduction of a series of signaling proteins and eventually cause a continuous increase in cytokine production (Figure 2).

### 3.1. IL-6

Il-6, which is known as the eye of the cytokine storm, performs a critical part in inflammatory and immunological responses. IL-6 transmits signals and exerts its effect by three important signaling modes, namely, the classic signal patterns, trans-signaling pathway, and trans-presentation pathway [36–38]. In the classic pathway, IL-6 adheres to the interleukin 6 receptor (IL-6R), which is also noted as GP80 via its site 1 to form a trimer. Subsequently, it interacts with another membrane glycoprotein (GP130) at site 2. As a consequence, a heterotrimer is formed. And then, an active hexameric signaling complex is formed in which GP130 initiates signal transduction through dimerization and phosphorylation by integrating two heterotrimers [39]. Downstream signal transduction of the hexameric signaling complex is mainly regulated by JAKs (Janus kinases) and STAT3 (signal transducer and activator of transcription 3). In addition, there are two pathways which also play a critical role in downstream signal transduction, namely, Akt-PI3K-MTOR (protein kinase B-phosphoinositol 3 kinase-mammalian target of rapamycin) and Ras-Raf-ERK-MAPK (GTPase-Raf kinase-extracellular signal-regulated kinase-mitogen-activated protein kinase) [40]. Although IL-6R is expressed only in a few cell types, IL-6 can also be transduced through connecting to soluble IL-6 receptor (sIL-6R) and subsequently to GP130 when it is at an elevated level. Current opinion holds that the IL-6 classic signaling pathway mainly exerts an important role in anti-inflammatory effects; on the other side, the trans-signaling pathway does the opposite; therefore, at high concentrations, IL-6 can both expand the scope of the inflammatory response and prolong the duration of activation of the pathway [1, 39]. In contrast to the classic pathway, the membrane-bound IL-6 receptor (mIL-6R) and GP130, which are mutually recognized in the trans-presentation pathway, are distributed in different cells: IL-6 combines with mIL-6R on dendritic cells and then binds to GP130 expressed on the membrane of T cells. This pathway plays a critical part in the activation of helper T cells 17 (Th17).

The IL-6-mediated JAK/STAT3 pathway is requested for complete stimulation of the NF-*κ*B pathway. Activation of NF-*κ*B can lead to IL-6 production, resulting in a cascaded amplification effect [41]. During this process, both IL-1 and TNF-*α* activate the NF-*κ*B pathway and promote IL-6 generation [42]. As a result, IL-6 promotes production of IL-1 and TNF-*α* through activating Th17 and other pathways in turn [43, 44]. Therefore, IL-6 performs a critical part in the activation of other cytokines.

### 3.2. TNF-*α*

TNF-*α* induces downstream effects mainly by activating the apoptotic pathway, NF-*κ*B pathway, and activator protein (AP-1). It is clear that TNF-*α* can interact with two types of receptors, which are TNF receptor 1 (TNFR1) and TNFR2. Researches show that TNFR1 has an intracellular death domain (DD) while TNFR2 does not [45]. The DD in TNFR1 can recruit TRADD. And then, TRADD recruits FADD, which can activate caspase-8 and caspase-3 sequentially, consequently causing cell apoptosis. Caspase-3 can also be activated by caspase-9, which requires mitochondria to release reactive oxide species (ROS) to mediate activation [46, 47]. The synergistic impact of TNF-*α* and IFN-*γ* can exacerbate the activation of caspase/FADD [46, 47]. In addition, TNF-*α* activates AP-1 through a variety of pathways, including ERK, p38MAPK, and C-JNK [47, 48]. AP-1 performs an important part in cell proliferation and death [49]. Although different from the apoptosis pathway, NF-*κ*B mainly mediates the expression of proteins related to cell proliferation and survival, playing an important proinflammatory role. For example, NF-*κ*B induces inducible nitric oxide synthase (iNOS). Furthermore, cyclooxygenase-2 (COX-2) and 5-lipoxygenase (5-LOX) are also upregulated [50, 51]. In addition, TNF-*α* not only exerts a proinflammatory effect through NF-*κ*B but also promotes the further spread of inflammation by upregulating the expression of cytokines. This process in reverse leads to upregulation of cytokines including TNF-*α*. In summary, TNF-*α* can both induce cell pyroptosis and apoptosis through caspase and AP-1 and promote the inflammatory response through NF-*κ*B.

### 3.3. IFN-*γ*

The host defense response mediated by interferon (IFN) mainly includes antiproliferative and proapoptotic effects. IFN mainly have three types in human [52]. Type I IFN refers to IFN-*β*, 13 types of IFN-*α* subtypes, and IFN-*ε* and IFN-*ω*. Type II IFN includes only one sort: IFN-*γ*. Type III interferons are found by researches so far, namely, IFN-*λ*1, IFN-*λ*2, IFN-*λ*3, and IFN-*λ*4. IFN-*γ* is the only type II interferon. Unlike other interferons, IFN-*γ* not only has antiviral properties but is also able to stimulate and modulate the immune system [53]. Similar to IL-6, IFN-*γ* recognizes the IFN-*γ* receptor (IFNGR1/2) and activates the JAK/STAT signal transduction pathway to perform its biological function [54, 55]. Subsequently, IFN-*γ* receptor activation results in STAT1 being phosphorylated by receptor-associated JAK1 and JAK2. A homologous dimer is formed by phosphorylated STAT1 that is transferred to the nucleus to activate the IFN-*γ* response gene by connecting to the GAS motif within the promoter [56]. The IFN-*γ* gene also has adhering sites which is in its promoter region for several other transcription factors. Among these transcription factors, AP-1, CREB/ATF, NFAT, NF-*κ*B, T-BET, and Eomes are included [57]. In addition, many transcription factors can be activated by other cytokines such as TNF-*α*, IL-1, IL-2, IL-6, IL-12, IL-15, IL-18, and IL-27. Type I interferon can also induce IFN-*γ* production by activating STAT-1 [58]. IFN-*γ* induces the stimulation of macrophages and Th1 cells, which activates the downstream cytokine network and maintains the continuous production of IFN-*γ*, leading to the activation of CD4^+^ and CD8^+^ cells [59–61]. IFN-*γ* performs a critical part in cell apoptosis stimulated by IFN regulatory factors (IRF). The IRF target genes responsible for the apoptotic response may include caspase-1, caspase-7, and caspase-8 [57].

### 3.4. IL-1 and the Inflammasome

IL-1 performs a significant role in inflammatory cascade amplification; to exert its effect during the innate immune response, it requires the processing and activation of a multiprotein complex called the inflammasome. The inflammasome can be divided into different types according to sensor proteins, including NLRP1, NLRP3, NLRC4, and AIM2 [62]. It serves as a regulator of caspase-1 stimulation and processing of IL-1*β*. The NLRP3 inflammasome has been found to play a key part in activating IL-1 to exert inflammatory effects [63]. The NLRP3 inflammasome consisted of NLRP3 and ASC (adaptor protein apoptosis-associated speck-like protein containing a caspase-recruitment domain) and effector protein inflammatory cysteine aspartase (caspase). Activation of the inflammasome is divided into two steps. When sensor proteins recognize DAMP or PAMP, NLRP3 interacts with ASC to form oligomers that provide a site for caspase-1 activation: this constitutes the first step. Caspase-1 activation also requires the mediation of the second signal in the second step. It is believed that the secondary signal may be derived from K^+^ efflux, mitochondrial DNA oxidation and dysfunction, lysosomal degradation, ROS formation, and Ca^2+^ imbalance [64, 65]. After two steps, caspase-1 is activated and promotes the excretion of IL-1*β* and IL-18.

In addition to cytokine production, the inflammasome can also cleave the connector site between the amino terminal (N) and carboxyl terminal (C) of Gasdermin D (GSDM-D), resulting in the formation of pores in the cell membrane through the N-terminal (GSDM-DN) [62]. This pore can release IL-1*β* and allow Na^+^ and H_2_O to pass into cells, resulting in cell swelling and apoptosis [66]. Excessive IL-1*β* not only promotes the secretion of a great number of cytokines (including IL-6, TNF, IFN-*α*, and IFN-*β*) through the stimulation of several signaling pathways but also activates cyclooxygenase, leading to acute inflammatory disease. Deposition of IL-1*α* can further activate PAMP or DAMP and promote the operation of the IL-1*β* pathway [67]. Recent studies have also found that other inflammasomes can increase caspase-1 activity through different pathways, causing similar inflammatory effects. Like caspase-1, several other proteins in the caspase family play important roles in inducing GSDM-D cleavage and apoptosis [62, 68]. For example, it is found recently that caspase-8 stimulates the secretion of proinflammatory cytokines and to process pro-IL-1*β* and IL-18 in identical routes as caspase-1 in inducing apoptosis.

## 4. The Mechanism of Organ Damage in Cytokine Release Syndrome

**Figure 3:**
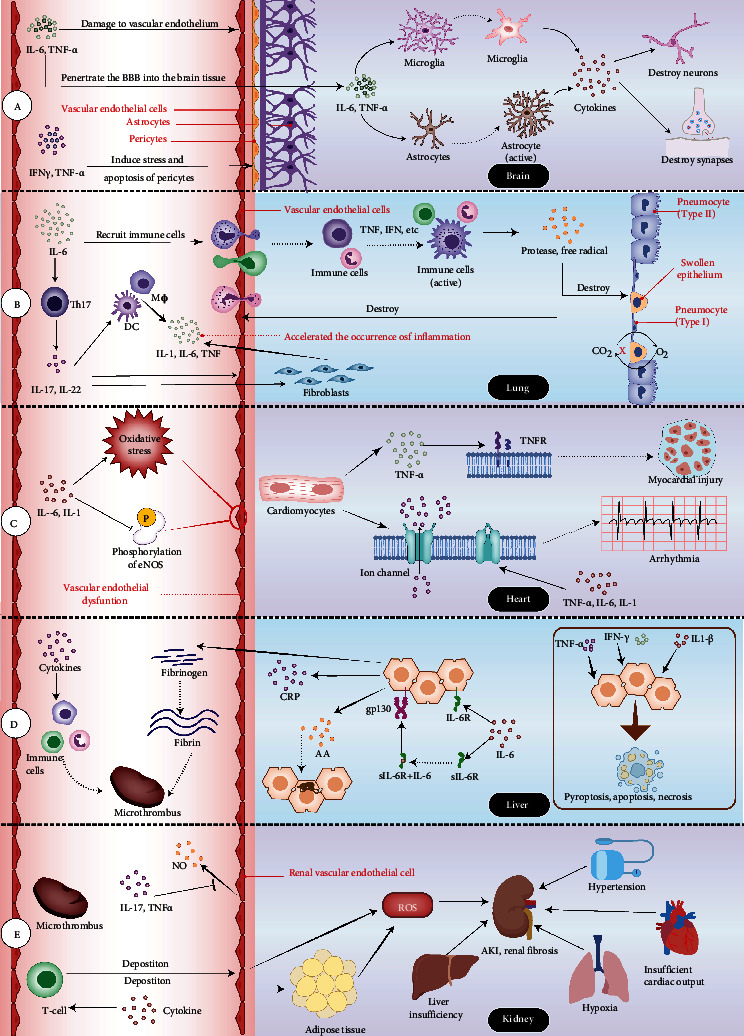
The mechanism of organ damage in cytokine release syndrome. (a) The mechanism of brain damage in cytokine release syndrome. IL-6 and TNF-*α* act on the vascular endothelium to increase the permeability of the BBB and activate the astrocytes and microglia to release cytokines, impacting the structure and function of neurons and synapses. (b) The mechanism of lung injury in cytokine release syndrome. Immune cells are recruited to the lungs by IL-6 and activated by cytokines such as TNF and IFN, generating huge amounts of free radicals and proteases, causing pulmonary edema and gas exchange problems. (c) The mechanism of heart damage in cytokine release syndrome. TNF-*α* and IL-6 can increase oxidative stress, reduce eNOS phosphorylation, and cause coronary endothelial dysfunction. Excessive TNF-*α* binding to receptors on cardiomyocytes can cause myocardial damage and dysfunction. TNF-*α*, IL-6, and IL-1 regulate the expression and function of ion channels on myocardial cell membranes, leading to arrhythmia. (d) The mechanism of liver injury in cytokine release syndrome. IL-6 binds IL-6R across the membrane to exert an anti-inflammatory effect. When IL-6 levels rise, it can combine with sIL-6R to promote inflammation and induce the production of acute phase proteins, culminating in microthrombosis and liver amyloidosis. (e) The mechanism of kidney injury in cytokine release syndrome. IL-17 and TNF-*α* work synergistically to suppress NO production in the vascular endothelium and enhance blood vessel contraction. Due to the chemotaxis of cytokines, T cells are deposited in blood vessels and enter tissues to produce ROS, leading to kidney damage and renal fibrosis. Microthrombi entering the renal capillary network can easily form microinfarction foci, leading to acute necrosis of renal tubules. Abbreviations: BBB: blood–brain barrier; eNOS: endothelial nitric oxide synthase; sIL-6R: soluble IL-6 receptors; NO: nitric oxide.

CRS involves damage to various organs throughout the body, mainly the brain, lungs, heart, liver, and kidney (Figure 3). Although these organs are connected to each other through blood vessels, the mechanism by which damage occurs to each of them is different.

### 4.1. Brain

A multitude of etiologies can cause neurological symptoms in cytokine release syndrome. Clinical studies have shown that increased blood–brain barrier (BBB) permeability can be observed in sufferers with neurological symptoms of CRS [69]. The appearance of neurological manifestations in COVID-19 patients with cytokine release syndrome-related encephalopathy is also associated with an increase in serum S100B protein levels (reflecting BBB permeability) [70, 71]; CAR-T cells, elevated protein concentration, and high white blood cell count have also been observed in the cerebrospinal fluid of patients with neurotoxicity after CAR-T cell adoptive immunotherapy [72]. Abnormal increase of cytokines in blood vessels can lead to increased BBB permeability. For example, IL-6 and TNF-*α* have been shown to cause endothelial damage and increase BBB dysfunction [73], allowing cytokines in the blood vessels to penetrate into the brain tissue through the BBB [72], subsequently activating the microglia [74] in the central nervous system to secrete IL-1*β*, IL-6, IL-12, TNF-*α*, and other cytokines [75], as well as activating other brain tissue cells such as astrocytes. Microglia and astrocytes can damage neurons and glial cells, which could explain the neurological symptoms seen in severe CRS. In addition, the passage of IFN-*γ* and TNF*α* through BBB can induce the pressure and apoptosis of vascular supporting pericytes, which may amplify the increase in BBB permeability and aggravate neurological symptoms [76, 77].

When cytokines in the blood increase the permeability of the BBB, viruses and CAR-T cells are also more likely to infiltrate and directly damage the brain tissue [72]. In addition, vascular leakage, complement activation, and abnormal coagulation caused by cytokine storms increase the risk of stroke and cause ischemic necrosis of brain tissue [22, 78]. It is worth noting that neurodegenerative diseases are also related to abnormal proinflammatory factors in brain tissue [79]. Therefore, CRS may worsen the condition of such patients while also increasing the risk of neurodegenerative diseases among other patients in the future: this warrants close attention from researchers.

### 4.2. Lung

Lung damage is very common in CRS, especially in severe respiratory infectious diseases, which can result in ARDS. The lung parenchyma is mainly composed of alveolar epithelial cells and vascular endothelium. In severe respiratory infections, the causative pathogen can lead to ARDS by recognizing the receptors of alveolar epithelial cells. In both infectious and iatrogenic cytokine storms, cytokines tend to accumulate in the lungs due to the abundance of small blood vessels in these organs.

Excessive IL-6 signaling can induce innate and adaptive immune cells to accumulate in the lungs [80]. When triggered by cytokines like TNF and IFN, these immune cells release large amounts of free radicals and proteases, causing damage to capillary endothelial cells and lung epithelial cells [17]. They can also act in concert with the inflammasome complex to promote the pyroptosis of alveolar cells [81]. A study has found that IL-6 can reduce the production of fibronectin [82], causing the connections between cells to loosen. Together with the edema and pyroptosis of alveolar and vascular epithelial cells, the permeability of the respiratory membrane (alveolar-capillary membrane) increases. Because IL-6 also damages liver cells, resulting in reduced albumin production, a great deal of fibrin-rich fluid and protein eventually leaks into the lung interstitium and alveoli, causing noncardiogenic pulmonary edema [83]. IL-6 can also promote the differentiation and maturation of Th17 cells, which produce IL-17 and IL-22, as well as promote the production of IL-1, IL-6, and TNF by macrophages, dendritic cells, fibroblasts, and endothelial cells, thereby accelerating the occurrence of inflammation and leading to lung tissue structure and dysfunction [43, 83]. Decreased lung function leads to hypoxia in tissue cells, which leads to decreased Na-K-ATPase activity of alveolar epithelial cells, cell metabolism disorders, lymphatic decompensation, and further aggravation of fluid retention and hypoxia. The oxygen-sensitive proline hydroxylase is activated and NF-*κ*B is released at this stage, which further aggravates the inflammatory response. Eventually, lung function deteriorates severely and ARDS develops [84].

### 4.3. Heart

The manifestations of the cardiovascular system in CRS include myocardial injury, myocarditis, arrhythmia, ischemic heart disease, and HF [23, 85]. In CAR-T treatment and COVID-19, CRS is intimately linked to the occurrence of heart damage and cardiovascular events [86, 87]. In specific CRS diseases, cardiac complications are often caused by various mechanisms, and abnormally increased cytokines are a major source of the secondary cardiac complications of the disease [85, 87]. Inflammatory cytokines mainly affect heart function in three ways: (1) acting on cytokine receptors on cardiomyocytes, causing structural changes in the heart; (2) acting on the specific ion channels of the ventricular myocytes, affecting the electrophysiological activity of the myocardium; and (3) acting on the endothelium of coronary arteries, affecting the blood supply to the heart.

Excess TNF-*α* may activate TNFR, causing myocardial dysfunction and infarction development, as well as hypertrophy, fibrosis, and apoptosis [88]. TNFR-*α* activation mediates cell apoptosis through the transduction of a series of downstream signals [17]. Studies have shown that IL-1, IL-6, and TNF-*α* can regulate the expression and functional changes of potassium and calcium channels on ventricular myocytes, which leads to prolonged duration of action potential of cardiomyocytes and affects the QT interval of cardiomyocytes [89–92]. This may be one of the mechanisms by which systemic inflammation in CRS leads to arrhythmia. Furthermore, in the mouse model, the interaction between TNF-*α* and IL-6 has been shown to aggravate oxidative stress and reduce eNOS phosphorylation, leading to coronary artery endothelial dysfunction in type 2 diabetes mellitus mice [93]. Coronary endothelial dysfunction can increase the occurrence of cardiovascular events [94], which may explain the frequent occurrence of cardiovascular complications when CRS occurs in patients with chronic underlying diseases.

It is worth noting that the mechanisms by which the above-mentioned inflammatory cytokines affect the structure and function of myocardium are chronic; as a result, not every patient with CRS will experience immediate cardiovascular symptoms. However, in patients with cardiovascular diseases and chronic diseases accompanied by inflammatory conditions such as diabetes, abnormally elevated inflammatory cytokines may aggravate the original heart damage, making these patients more likely to develop CRS-related cardiovascular events.

### 4.4. Liver

The liver plays a key role in purifying the blood and removing harmful substances because besides liver cells there are a great deal of other immune cells in the liver such as Kupffer cells, dendritic cells, and lymphocytes. Damaged liver cells thus interact with these immune cells. Clinical studies have shown that cases of liver damage can be found in a variety of diseases exacerbated by CRS. Damage of liver cells is closely related to cytokine storms, in which IL-1, IL-6, TNF-*α*, and IFN all play a role.

IL-6 can exert its effect in two ways: classic and trans-signaling. Studies have found that the classic signaling pathway through IL-6R that bound in membrane is required for anti-inflammatory effects and can promote the regeneration of liver and biliary tract cells. When the concentration of IL-6 increases, it can combine with sIL-6R to exert its proinflammatory effect and readily induce the production of acute phase proteins like serum amyloid A (SAA), C-reactive protein (CRP), haptoglobin, and fibrinogen [82]. The accumulation of amyloid (AA) can lead to amyloidosis in the liver, and in severe cases, the very poor outcome of liver failure can be observed [95]. The deposition of AA can also affect the kidneys and gastrointestinal tract, leading to multiple organ failure [95]. IL-6 induces insulin resistance and causes fatty changes in liver cells [96]. The acute phase protein and fibrin participate in the activation of the complement system and the coagulation cascade, leading to a persistent hypercoagulable state of blood. Cytokines recruit monocytes, neutrophils, and other immune cells to the vascular endothelium, and the complement system and procoagulant pathways interact, leading to the formation of microthrombi [97–99]. Liver dysfunction can also disrupt the coagulation and anticoagulation balance and induce DIC in severe cases.

IL-1 produces IL-1*β* after the activation of inflammasomes, which not only induces the pyroptosis of hepatocytes but also activates other cytokines in the liver to form a positive feedforward stimulus and continue to amplify inflammatory damage. Several human and animal experiments have confirmed that the inflammasome and IL-1 are the main factors that drive liver cell damage and liver failure [100]. The liver is rich in NK cells, which account for about 30% of immune cells [101–103]. A study showed that under normal circumstances, IFN-*γ* secreted by NK cells can promote liver regeneration after activating STAT3. Overactivated NK cells can activate STAT1 signaling pathway in an IFN-*γ*-dependent manner and block the proliferation of hepatocytes and oval cells to inhibit liver regeneration [104]. TNF-*α* plays a dual role in the liver. It not only acts as a cell death mediator but also induces hepatocyte proliferation and liver regeneration. TNF-*α* can prevent cell death through the NF-*κ*B pathway and induce hepatocyte apoptosis and necrosis through the ROS-JNK pathway [105].

### 4.5. Kidney

There are many reasons for kidney damage caused by cytokine storms. Hypertension, immune cell recruitment, and microthrombosis are all potential contributors. The dysfunction of other organs can also affect the kidneys. IL-6 promotes the differentiation and maturation of Th17, and the synergistic effect of secreted IL-17 and TNF-*α* reduces NO production in the vascular endothelium and enhances blood vessel contraction; the release of IFN-*γ* regulates the production of local angiotensinogen in the kidney; subsequently, the AngII pathway and the renin-angiotensin-aldosterone system (RAAS) are overactivated, and the production of aldosterone increases, which enhances the reabsorption of water and sodium and aggravates high blood pressure [106–110]. Hypertension damages the renal capillary endothelial cells, leading to renal arteriole sclerosis. T cells deposited in renal capillaries due to the chemotaxis of cytokines penetrate into the adventitia of the capillaries and surrounding fat to produce ROS, leading to kidney damage and renal fibrosis [110]. Severe hypercoagulability can lead to diffuse intravascular coagulation, and microthrombi entering the renal capillary network can form renal microinfarction foci, leading to acute necrosis of renal tubules and even affecting cortical function [111].

In the late stage of CRS, if the heart experiences cardiac insufficiency due to cytokine storms, insufficient cardiac output may lead to insufficient renal perfusion [112]. Lung damage leads to hypoxia, while liver insufficiency leads to hepatorenal syndrome, which also contributes kidney damage.

## 5. Treatment

There are several types of drugs that can be used to prevent the development of CRS; these include cytokines or cytokine receptor antagonists, downstream signaling pathway blockers, glucocorticoids, and plasma from recovered patients (Table 1).

### 5.1. Cytokine Antagonism

#### 5.1.1. IL-6 Antagonists-Clazakizumab

Clazakizumab is a high-affinity humanized monoclonal antibody developed by genetic engineering (IgG1). It prevents IL-6 from interacting with IL-6R. Clazakizumab has the best effect and longest acting time among the IL-6- and IL-6R-blocking drugs. It does not cross-link with any cell surface receptors and has no antibody-dependent or complement-dependent cytotoxicity. In addition, compared with IL-6 receptor blockers, clazakizumab can avoid the rebound effect caused by accumulated IL-6 [113]. According to a case report, a severe COVID-19 patient who had received a heart transplant was cured after receiving clazakizumab [114]. Clazakizumab has also been used for treating rheumatoid arthritis, significantly improving the response rate of ACR20 compared with that in patients treated with methotrexate (MTX) alone [115]. Another study has shown that clazakizumab can reduce donor-specific antibody (DSA) titers in the immune rejection after kidney transplantation and significantly improve the prognosis of kidney transplantation patients [116]. Other drugs in the same category are olokizumab [39], siltuximab, and sirukumab.

#### 5.1.2. IL-6 Receptor Antagonist-Tocilizumab

Tocilizumab is a humanized monoclonal antibody which can bind to soluble IL-6R (GP80) and membrane-bound IL-6R (GP130); it blocks the IL-6 signal pathway to reduce inflammation and prevent the progression of CRS. Data from a retrospective study conducted in China [117] showed that most of the severe COVID-19 patients and critical COVID-19 patients experienced immediate improvement in hypoxemia and lung CT opacity after tocilizumab treatment, which indicates that tocilizumab has a great treatment of COVID-19. Its mechanism and efficacy in rheumatoid arthritis are similar to those of clazakizumab [118]. Other drugs in the same category are sarilumab and levilimab [119, 120].

#### 5.1.3. Blockade of IFN-*γ-Emapalumab*

Emapalumab is a monoclonal antibody against IFN-*γ*. It binds to the cytokine IFN-*γ* to block its interaction with the corresponding cell surface receptors and prevents its activation of downstream JAK/STAT-related signaling pathways, thus preventing the further aggravation of CRS. It can significantly reduce critical COVID-19 patients' mortality. However, its side effect is to increase the patients' viral load and exacerbate the infection [121]. This drug blocks cytokine storms in primary hemophagocytic lymphohistiocytosis (pHLH) [122], and it is currently approved by the US FDA (Food and Drug Administration) for the treatment of the disease.

#### 5.1.4. Blockade of IL-1*β-Canakinumab/Anakinra*

Anakinra is an IL-1 receptor antagonist. Canakinumab is an IL-1*β* antagonist. These drugs prevent NLRP3 activation by blocking the binding of IL-1 and its receptor, preventing apoptosis and pyroptosis, and reducing induction of the release of other cytokines by the IL-1 pathway to prevent the further development of CRS. Studies have shown that anakinra treatment can significantly reduce mortality in COVID-19 patients without increasing the risk of infection and with no side effects. Anakinra is therefore an ideal broad-spectrum therapeutic drug [123, 124]. In a recent study, 10 hospitalized adults with bilateral pneumonia, excessive inflammation, and respiratory failure who did not require mechanical ventilation showed a rapid decrease in serum CRP, improved oxygenation, and no granulocytopenia or bacterial septicemia during 45 days of hospitalization when treated with canakinumab [125].

#### 5.1.5. Blockade of TNF-*α-Infliximab/Adalimumab/Etanercept*

This type of drug is a soluble TNF-*α*-binding drug, which prevents the binding of TNF-*α* to its receptors and activates downstream signaling pathways. Some researchers have suggested that treatment with high doses of infliximab may worsen infection, but existing clinical studies do not offer enough evidence to support this claim. Anti-TNF therapy has been shown to be safe for many people at high risk of COVID-19 [126]. Furthermore, studies have shown no direct association between the drug and adverse pregnancy outcomes [127]. Etanercept is a humanized TNF-*α* receptor antibody fusion protein, a soluble TNF receptor, which prevents the binding of TNF-*α* and its receptor by binding to TNF-*α*/*β* to control inflammation. Because its mechanism of action is similar to infliximab and adalimumab, the authors believe that this drug can be used to treat COVID-19; however, this needs verification through relevant clinical trials.

### 5.2. Blockade of Cytokine Downstream Pathway

#### 5.2.1. JAK-STAT Signaling Pathway-Ruxolitinib

Ruxolitinib is a potent inhibitor of the JAK/STAT signaling pathway that selectively inhibits JAK-1 and JAK-2 to exert antiviral effects. Ruxolitinib inhibits the function of T cells, dendritic cells, and NK cells to reduce the production of IL-10, IL-12, IL-23, and TGF-*β*. Reduced IL-12 and IL-23 levels result in the inactivation of Th-1 and Th-17 cells. This reduces the production of IL-17, IL-22, TNF-*α*, IFN-*γ*, and IL-2, ultimately weakening the inflammatory response [128]. However, this downregulation increases the body's susceptibility to opportunistic pathogens and the risk of activation of latent infections. However, a number of controlled clinical studies [129, 130] have shown that the use of ruxolitinib in COVID-19 patients can accelerate clinical improvement and is well tolerated.

#### 5.2.2. NF-*κ*B Signaling Pathway-Phillyrin (KD-1)

In laboratory studies, phillyrin was shown to significantly reduce the levels of NF-*κ*B, thereby reducing the production of inflammatory cytokines such as IL-6, TNF-*α*, IL-1*β*, IP-10, and MCP-1 to relieve inflammation [131]. However, there is a lack of studies related on its clinical application for COVID-19 treatment.

#### 5.2.3. PPAR-*γ* Agonists

Thiazolidinedione (TZD) and pioglitazone have attracted much attention [132, 133]. PPAR-*γ* (peroxisome proliferator-activated receptors-*γ*) is widely distributed in tissues. It not only inactivates NF-*κ*B by silencing but also induces the synthesis of antioxidant enzymes and reduces the generation of ROS [134, 135]. Furthermore, PPAR-*γ* inhibits the differentiation and maturation of monocyte macrophages [136]. To date, many reviews have hypothesized that PPAR-*γ* drugs have a good therapeutic effect in CRS, reducing lung injury and inhibiting viral RNA synthesis and replication [137].

#### 5.2.4. SphK-S1P-S1PR

Current FDA-approved drugs targeting SphK-S1P-S1PR include FTY720, ozanimod, and opaganib. Activation of FTY720 leads to downregulation of S1PR_1_ and reduces microvascular permeability, thereby preventing the invasion of lymphocytes from lymphatic tissue into the blood system, which is instrumental in limiting the diffusion of inflammation [138, 139]. FTY720 inhibits the generation of TNF-*α*, IL-6, and other types of cytokines [140], thus taking part in suppressing cytokine storms. Additionally, clinical studies have found that FTY720 is able to pass through the BBB as well as fulfill multiple roles in the CNS. Since more serious neurological side effects may occur in patients with COVID-19, it may be utilized as an early prevention measure [141, 142]. Compared with FTY720, ozanimod has the advantage that it does not bind to S1PR_3_ and activate it. Hence, ozanimod is more specific, having fewer cardiopulmonary side effects. Furthermore, it has a shorter half-life as well as higher safety [143]. In global clinical trials for patients infected with SARS-COV-2, opaganib has also been measured. Some studies suggest that sphingolipid derivatives, such as ceramide-1 phosphate, have stronger potential of antiviral and auxiliary immune in COVID-19 [144–146].

### 5.3. Other Treatment

In addition to the therapeutics described above, the use of glucocorticoid (dexamethasone) has been shown to be effective in reducing 28-day mortality in COVID-19 patients without significantly increasing the risk of adverse events [147]. In China, during the COVID-19 outbreak, traditional Chinese medicine has also played a significant role in improving the prognosis of patients and accelerating their recovery. A multicenter controlled study [148] conducted by academician Zhong Nanshan's team showed that luteinizing hormone capsule (Lianhuaqingwen) improves the recovery rate of patients with fever, fatigue, cough, and other symptoms and shortens their recovery time. Lianhuaqingwen has been approved for COVID-19 treatment in China. In Wuhan, plasma treatment of recovered patients also achieved good results. A small clinical study has shown that treating COVID-19 patients with high-titer convalescent plasma results in remission or resolution of clinical symptoms such as fever, cough, shortness of breath, and chest pain within 1–3 days. Lung imaging findings and lung function were also reported improving significantly [149]. This also suggests that pathogen control may be a treatment for cytokine release syndrome given that the persistence of pathogens is an important factor in maintaining cytokine storms. Some researchers believe that high doses of vitamin C can also play a role in blocking cytokine storms [150].

## 6. Discussion

We note that patients with the same CRS disease still have different clinical manifestations; this is evident when COVID-19 patients are considered as an example. COVID-19 patients are classified based on whether they have asymptomatic, mild, moderate, severe, or critical disease. Some patients develop ARDS and die. The difference in clinical manifestations may be due to the difference in the negative feedback regulation mechanism of inflammatory response among different populations. The imbalance between the virus's ability to cause inflammation and the patient's ability to suppress it leads to disease progression and even death. Plasma treatment can quickly relieve the clinical symptoms: a possible explanation for this is that in COVID-19 survivors, the virus-specific antibody in the blood plasma can neutralize the virus directly and undermine its ability to promote the progression of inflammation to levels below which the patient's own immune system can inhibit inflammation progression quickly; in this way, the patient's own negative feedback mechanism can be brought into play to alleviate clinical symptoms. However, the specific mechanism of action of convalescent plasma therapy has not been fully elucidated.

IL-6, IFN- *γ*, TNF- *α*, and IL-1 participate in the activation of multiple key pathways in the cytokine network simultaneously, among which extensive studies refer to the NF-*κ*B pathway, JAK/STAT pathway, and MAPK pathway [41, 42]. Therefore, these four cytokines are closely related to a variety of other different kinds of cytokines. Because of the diverse combinations of cytokine receptors and signal transduction pathways, one signaling molecule may not only participate in one pathway but also affect the transduction of other pathways. Therefore, single-target drugs can theoretically have high efficacy in a short time, but it cannot be ruled out that other signaling pathways may be affected which could generate potential side effects such as disruption of the trade-off between the process of proinflammatory and anti-inflammatory. Studies of single-target drugs should focus on the overall impact of blockers on upstream and downstream pathways to identify such side effects as priority.

At present, in addition to the interaction between cytokines, other components in tissues play key roles in the activation or inhibition of cytokines. For example, the activation of the inflammasome requires second signals, including second messengers. Besides the binding of DAMPs and PAMPs with PRRs, the changes in intracellular ions and the dysfunction of mitochondria and lysosomes also play important part in inflammasome activation [64, 65]. Therefore, the mechanism of action of these components in CRS is also worthy of further exploration.

The increased vascular permeability and endothelial dysfunction is one of the crucial shared mechanisms of multiple organ dysfunction in CRS. However, the characteristics of mechanism by which organ damage occurs in CRS vary in different organs such as the brain, lung, heart, liver, and kidney, giving rise to divergent future research directions. For example, the symptoms of neurological damage in CRS are more common in patients receiving CAR-T treatment, while symptoms of respiratory system damage in CRS are more common in severe infectious diseases of the respiratory tract, indicating that the degree of organ damage is related to the cause. In CRS, IL-6 plays a central role in the mechanism of pulmonary injury, TNF-*α* plays a vital role in the mechanism underlying cardiac injury, and the liver injury mechanism includes a variety of cytokines, indicating that the contribution of cytokines to damage in different organs is different. The damage to the liver plays an important role in aggravating multiple organ failure in CRS, while kidney damage is more secondary to the damage to other organs, indicating that different organ disorders play different roles in CRS. In addition, the influence of inflammatory factors on various systems plays an important role in the mechanism of multiple organ dysfunction in CRS, although the distribution of inflammatory factor receptors in various organs and their functions are not clearly understood. Furthermore, organ damage is often the result of the synergistic effect of multiple inflammatory factors, which also makes research challenging. Future research should pay attention to the effects of cytokines on specific organs and the interaction between cytokines.

Most of the functional proteins in the cytokine pathway are located in cells and some in the nucleus. Thus, in addition to extracellular specific factor antagonists, the development of key protein antagonists in shared intracellular or nuclear pathways is a significant orientation in developing therapeutic drugs against CRS. It is necessary to test the transmembrane ability of drugs and the efficiency and activity of cell entry when developing such drugs to better assess their efficacy. Because a variety of kinases is involved in the activation and signaling of transduction proteins, enzyme activity is also worthy of further investigation. At present, good results have been achieved in the targeting of pathogens, cytokines, cytokine receptors, and downstream signaling pathways. However, due to the multipathway characteristics of CRS itself, an effective specific drug or drug combination remains elusive. Although Chinese medicine also shows efficacy in the treatment of CRS-related diseases, there are still many uncertainties due to the unclear mechanism of action. We believe that as the mechanism of CRS is gradually clarified, more effective drugs and treatment plans will be developed. However, vaccination remains the most efficacious response to curb the burden of COVID-19.

## 7. Conclusion

Cytokine storms occur in many diseases and are a key determinant of poor prognosis. CRS, which is the result of the accumulation of cytokine storms in local tissues or organs, involves the mobilization of a great number of immune cells to attack and injure cells in tissues, causing structural destruction and dysfunction of tissues and organs. Prominent and common pathophysiological features include vascular leakage and necrosis. CRS involves a variety of cytokines; among which IL-6, IFN-*γ*, TNF-*α*, and IL-1 perform an extremely key role in the cytokine network, determining the severity of cytokine release syndrome. Although several drugs targeting specific cytokines have been used in some CRS-related diseases, it is uncertain whether these drugs have good efficacy against multiple CRS-related diseases. Therefore, elucidation of the pathophysiological mechanisms of these four cytokines in multiple organ dysfunction may reveal new ideas and directions for the treatment of cytokine release syndrome.

## Figures and Tables

**Table 1:** Drugs commonly used to inhibit cytokine storms.

Distribution	Representative drug	Targets	CRS-related diseases	Effect	Regulatory approval status
Cytokine/cytokine receptor antagonist	Clazakizumab	IL-6	Rejection of kidney transplantation COVID-19 Rheumatoid arthritis	DSA↓ IL-6↓ ACR20 response rate↑	Clinical trial
	Tocilizumab	IL-6R	COVID-19 Rheumatoid arthritis	All-cause mortality rate↓ ICU occupancy↓ ACR20/50/70 response rate↑	FDA
	Emapalumab	IFN-*γ*	pHLH COVID-19	IFN-*γ*↓	FDA
	Canakinumab/Anakinra	IL-1/IL-1R	Macrophage activation syndrome, rheumatoid arthritis, COVID-19	IL-1*β*↓ Clinical symptoms are relieved or under control	FDA
	Infliximab/adalimumab/ etanercept	TNF-*α*	COVID-19 Inflammatory bowel disease	TNF-*α*↓ IL-1↓ IL-6↓	FDA

Downstream pathway blockers	Ruxolitinib	JAK-1, JAK-2	COVID-19	IL-10/IL-12/IL-23/TGF-*β*↓	FDA
	Phillyrin	NF-*κ*B	COVID-19	Expression of proteins related to NF-*κ*B↓, IL-6↓, TNF-*α*↓, IL-1*β*↓, IP-10↓, and MCP-1↓	Clinical trial
	Thiazolidinedione	PPAR-*γ*	Type 2 diabetic nephropathy	Inhibition of NF-*κ*B	Clinical trial
	Fingolimod	S1PR	COVID-19	S1PR-1↓ TNF-*α*↓ IL-6↓	FDA

Other treatment	Dexamethasone	/	COVID-19 and systemic immune disease	Inhibits a variety of cytokines	FDA
	Lianhuaqingwen capsule	/	COVID-19	Alleviates clinical symptoms and shortens recovery time	NMPA
	Vitamin C	/	COVID-19	Inhibition of NF-*κ*B IL-6↓ TNF-*α*↓ GM-CSF signaling response↓ Reduces oxidative stress	/
	Plasma of convalescent patients	SARS-COV-2	COVID-19	RNA of COVID-19↓	FDA (EUA)

FDA: Food and Drug Administration (America); NMPA: National Medical Products Administration (China); EUA: Emergency-Use-Administration; pHLH: primary hemophagocytic lymphohistiocytosis; DSA: donor-specific antibody; IL-6/IL-1: interleukin-6/interleukin-1; IL-6R/IL-1R: interleukin-6 receptor/interleukin-1 receptor; IFN-*γ*: interferon-*γ*; ACR20: American College of Rheumatology 20%, a composite measure defined as both improvement of 20% in the number of tender and number of swollen joints and a 20% improvement in three of the following five criteria: patient global assessment, physician global assessment, functional ability measure (most often Health Assessment Questionnaire (HAQ)), visual analog pain scale, and erythrocyte sedimentation rate or C-reactive protein (CRP); TNF-*α*: tumor necrosis factor-*α*; JAK-1/JAK-2: Janus kinase-1/Janus kinase-2; NF-*κ*B: nuclear factor kappa-B; PPAR-*γ*: peroxisome proliferator-activated receptors; S1PR: sphingosine-1-phosphate receptor; SARS-CoV-2: severe acute respiratory syndrome coronavirus 2; GM-CSF: granulocyte-macrophage colony-stimulating factor.
